# Optimization of Lignin-Based Biocatalyst Production from Pine Sawdust and Wheat Straw

**DOI:** 10.3390/molecules23081877

**Published:** 2018-07-27

**Authors:** Froylán M.E. Escalante, Alejandra Carranza-Hernández, Adelina García-Zamora, Efrén Aguilar-Garnica

**Affiliations:** 1 Department of Chemistry, Universidad Autónoma de Guadalajara, Zapopan 45129, Mexico; froymario@edu.uag.mx (F.M.E.E.); carranzahernandeza@gmail.com (A.C.-H.); lina_zamora94@gmail.com (A.G.-Z.); 2 Mexican Bioenergy Innovation Centre, Bioalcohols Cluster, Av. del Bosque 1145, El Bajío, Zapopan 45019, Mexico

**Keywords:** Lignin, sawdust, wheat straw, sulfonation, catalyst, esterification, Box-Behnken

## Abstract

Pine sawdust and wheat straw are abundant lignocellulosic wastes that have been recently converted into bioethanol under a biochemical platform scheme whose main waste is lignin. Lignin can be transformed into a wide variety of high added-value products, including its functionalization as a catalyst. A key step in the synthesis of a lignin-based catalyst is the sulfonation reaction, whose operating conditions, namely, H_2_SO_4_ to lignin ratio (mL/g), temperature and time, have been arbitrarily chosen. In this contribution, an optimization methodology (i.e., Box-Behnken) is applied in order to found the operating conditions during the sulfonation reaction that maximizes the total acid sites density of lignin-based catalysts from pine sawdust and wheat straw. The optimization results show that the time in sulfonation reactions can be significantly reduced, compared to those previously reported, without affecting the performance of both catalysts in esterification reactions. These results could be further considered for energy and costs reduction purposes during the conceptual design engineering of the sulfonation reaction.

## 1. Introduction

Pine sawdust and wheat straw are the main wastes from pine and wheat’s (*Triticum* spp.) industrial processing, respectively. In Mexico, pine sawdust is produced at a rate of 1 × 10^5^ t per year [1], while wheat straw is generated at a rate of 4.6 × 10^6^ t per year [2]. These wastes have been usually exploited as low-cost products. For instance, pine sawdust has been considered as adsorbent for the disposal of olive mill wastewater [3] or as a fluoride remover [4], whereas wheat straw has been used for animal bedding [5] or as a cattle feed [6]. Recently, these wastes have also been considered as feedstock for (second generation) bioethanol production in a biochemical platform scheme [7,8], in which the hemicellulose and cellulose contained in such wastes are transformed into bioethanol, while the lignin content (i.e., residual lignin) is currently discarded or converted into electricity [9]. With respect to this case, a number of efforts have been made to convert residual lignin into added-value products, such as resins, carbon fibers, fertilizers, etc. [10]. However, little has been done to transform residual lignin into a high-value product, such as a catalyst. For instance, Pua et al. [11] have considered Kraft lignin powders for the synthesis of a lignin-derived catalyst, whose activity was assessed in the esterification of oleic acid. Besides, Guo et al. [12] have used the hulls of *Xanthoceras Sorbifolia* for the synthesis of a lignin-based catalyst in the esterification of soybean soapstock. Furthermore, Hu et al. [13] have synthesized a lignin based catalyst from activated carbon fibers for cellulose hydrolysis and, recently, Zhu et al. [14] have also catalyzed the hydrolysis of microcrystalline cellulose with a biocatalyst, whose structure is based on Masson pine alkali lignin. A common and key step during the synthesis of catalysts in the aforementioned contributions is the sulfonation reaction because it provides the catalytic sites (e.g., -SO_3_H group) to the lignin network. Nevertheless, there is a lack of consensus regarding the most recommended operating conditions to conduct the sulfonation reaction. In addition, to the best of our knowledge, there is no study devoted to the adequate selection of such operating conditions. Thus, this work was intended as a description of how a Box-Behnken design might be applied to elucidate the sulfonation reaction operating conditions: The sulfuric acid to lignin ratio, temperature and time that optimizes (i.e., maximizes) the number of total -SO_3_H groups (i.e., total acid sites density) in lignin-based catalysts from pine sawdust and wheat straw. Then, this paper contributes to the valorization of residual lignin coming from the biochemical scheme processing of abundant wastes through the optimization of biocatalysts synthesis.

The results concerning the optimization of the lignin-based catalyst synthesis from pine sawdust are still patent-pending in México (MX/a/2016/011129), while the results related to the catalyst from wheat straw will also be patented. This is why we are only presenting, in this paper, a partial characterization of both catalysts. The qualitative characterizations have been performed via Fourier Transform Infrared (FTIR) and the quantitative characterizations have been conducted by measuring the total number of -SO_3_H groups. Nevertheless, the lignin extraction process from pine sawdust and wheat straw, the transformation of these lignins to biocatalysts and the activity test of such biocatalysts in the esterification of oleic acid are fully detailed in the Materials and Methods section. Subsequently, the Box-Behnken design results are shown and expressed as the total number of -SO_3_H groups (i.e., total acid sites) as a function of the sulfonation reaction operating conditions. Finally, we discuss our results and compare them with those available in the literature within the Discussion section.

## 2. Results

### 2.1. Biocatalyst from Pine Sawdust

**Figure 1:**
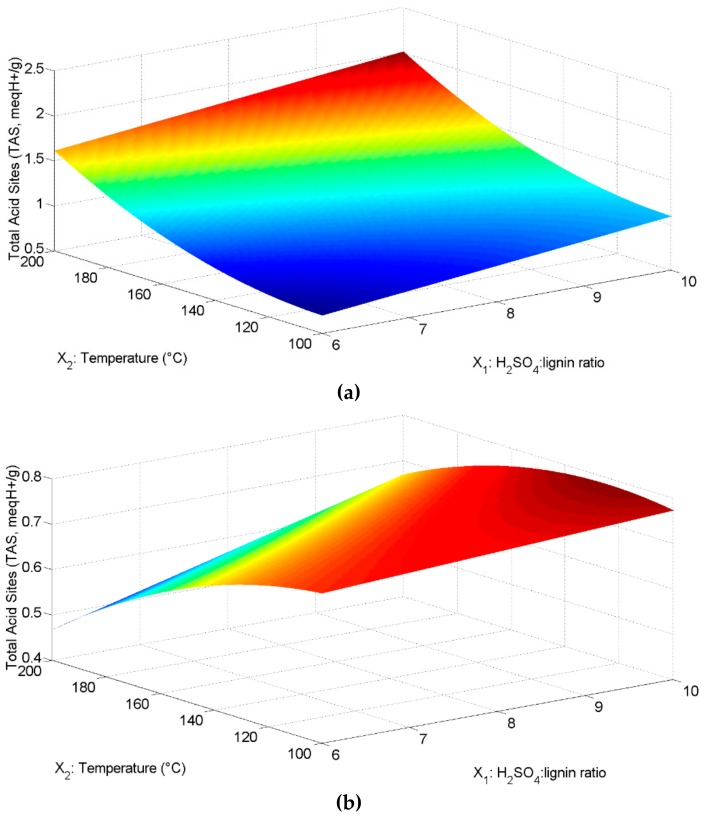
Surface responses for sulfonation experiments. (**a**) Surface response for sulfonation experiments of lignin from pine sawdust (time was set to the optimal value: 1 h); (**b**) Surface response for sulfonation experiments of lignin from processed wheat straw (time was set to the optimal value: 2 h).

The lignin extraction yield for the pine sawdust without ashes was 29.96 ± 1.47% (m/m) and, after computing the pyrolysis and the sulfonation yields, it was possible to reach 22.1% (m/m), as the global yield of biocatalyst synthesis. The numerical results of the experimental design are depicted in Table 1 for the sulfonation process, expressed as Total Acid Sites (TAS), whose factors were X_1_: Ratio of concentrated H_2_SO_4_ to lignin (mL/g), X_2_: Temperature (°C), and X_3_: Time (h). These results, depicted in Figure 1a, are in the form of a surface response whose mathematical model is given by

TAS = −1.004 + 0.378X_1_ − 0.007X_2_ + 0.947X_3_ − 0.279X_1_X_3_ + 0.0000864X_2_^2^ − 0.009X_2_X_3_ + 0.974X_3_^2^ (1)

**Figure 2:**
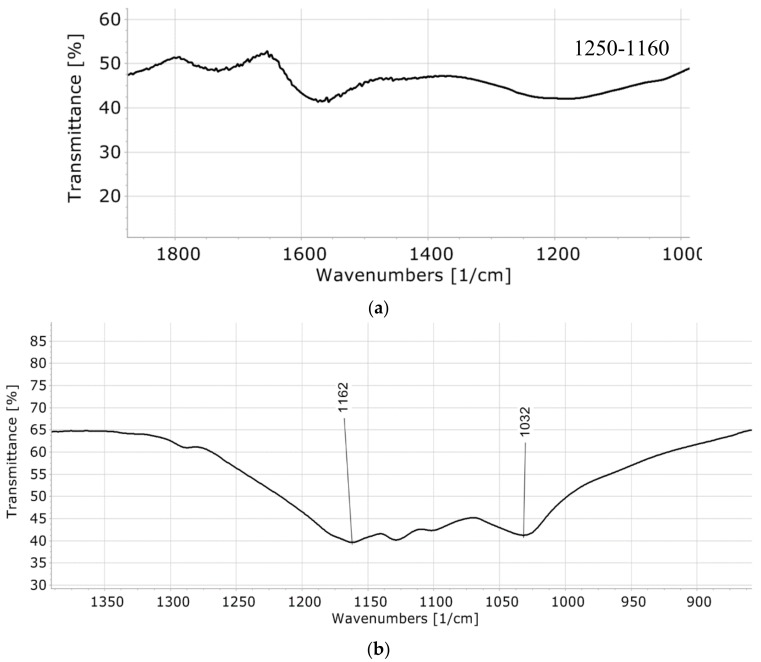
FTIR spectrum of biocatalysts. (**a**) FTIR spectra of the lignin-based biocatalyst from pine sawdust; (**b**) FTIR spectra of the lignin-based biocatalyst from processed wheat straw.

According to the ANOVA (Table 2), temperature (X_2_) and time (X_3_) were significant in such a design, with 0.95 confidence (*p* < 0.05). The interaction between the H_2_SO_4_-lignin ratio (X_1_) and time (X_3_) has a negative impact on the TAS (with *p* = 0.0006), and the same is true for the interaction between the temperature and time, but in a lesser degree (with *p* = 0.0027). A negligible positive effect can be observed for the squared term of temperature (X_2_^2^); however, it can be observed that increasing the time could increase the TAS by up to 4 units when the time is adjusted to 2 h (*p* < 0.0071). The three-way interaction of the factors was not considered, since it was not possible to analyze its effect. The predicted maximum TAS was 2.0 meq H^+^/g, considering a 10:1 (mL/g) H_2_SO_4_-lignin ratio, 200 °C and 1 h. However, when the biocatalyst was prepared accordingly, the obtained TAS was equivalent to 1.68 meq H^+^/g. The difference between the experimental and predicted values for TAS could be attributed to the determination coefficient of the model, which is 80.23%. This biocatalyst was characterized via FTIR (see Figure 2a) and used to promote the esterification reaction of oleic acid, with methanol achieving a reaction yield of 94.73 ± 0.63%.

### 2.2. Biocatalyst from Wheat Straw

The lignin extraction yield for the (processed) wheat straw was 32.85 ± 7.8% (m/m). Then, after computing the pyrolysis and sulfonation yields, it was determined that the global yield for the biocatalyst synthesis was 18.8% (m/m). The numerical results of the experimental design for the sulfonation process are also shown in Table 1. These results, depicted in Figure 1b, are in the form of a surface response whose mathematical model is given by

TAS = 1.013 − 0.029X_1_ + 0.002X_2_ − 0.559X_3_ + 0.0004X_1_X_2_ − 0.00002X_2_^2^ − 0.0005X_2_X_3_ + 0.216X_3_^2^ (2)

In this case, ratio of concentrated H_2_SO_4_ to lignin (X_1_) and temperature (X_2_) were significant in such a design, with 0.95 confidence (*p* < 0.05), according to the ANOVA, shown in Table 3. The interaction between the H_2_SO_4_-lignin ratio (X_1_) and temperature was slightly important, but significant (*p* = 0.0156); neither the time nor the interaction temperature–time were significant. Since the coefficient of the squared term, including temperature (X_2_^2^), is so small, its effect in the TAS could be almost neglected, even when it was significant (*p* = 0.0128); on the other hand, the squared value for the time (X_3_^2^) implies that increasing the time could increase, by up to 1 unit, the TAS every 2 h of reaction. The interaction, X_1_X_2_, implies an increment in TAS of about 1 unit when the sulfuric acid to lignin ratio is kept at 10:1 and the temperature at 200 °C; however, the interaction temperature–time has a slightly negative impact on TAS, decreasing it by 0.2 units when the highest values of time and temperature were assayed.

The predicted maximum TAS was 0.78 meq H^+^/g, considering a 10:1 (mL/g) H_2_SO_4_-lignin ratio, 125 °C and 2 h. As expected, these conditions differ from those obtained in the synthesis of the lignin-based pine sawdust catalyst. This is probably due to the different nature of lignins. Furthermore, when the biocatalyst was prepared accordingly to the conditions, predicted to be the best using the model, the obtained TAS was equivalent to 0.74 meq H^+^/g (i.e., it was very close to that predicted by the surface response, and this is probably because the determination coefficient for this model (88.15) is higher than that obtained for the pine sawdust). This biocatalyst was characterized via FTIR (see Figure 2b) and used to catalyze the esterification reaction of oleic acid with methanol, achieving a reaction yield of 88.49 ± 0.57%.

## 3. Discussion

Lignin content in pine sawdust (29.96 ± 1.47%) is similar to that reported by Shulga et al. [15], whereas lignin content in processed wheat straw (32.85 ± 7.8%) is higher than that reported by Kumar et al. [16]. This could be due to the nature of wheat straw, whose major part of sugars had been removed, leaving behind the lignin in higher proportion. In addition, the maximum TAS for the lignin-based biocatalyst from pine sawdust, using the optimal conditions, was 1.68 meq H^+^/g, and it was achieved after 1 h of the sulfonation reaction. This value in acid sites density is similar to that reported by Pua et al. [11] and Guo et al. [12], although they considered 2 h of sulfonation reaction time. This is also close to the values depicted in the studies carried out by Liang et al. [17] and by Adhikari et al. [18], unless they inverted 5 h and 12 h, respectively. Despite the lower number of acid sites, the biocatalyst is almost equally efficient in the esterification reaction than those previously cited, although our biocatalyst was synthesized, investing only 1 h of the sulfonation reaction. Furthermore, the FTIR spectra of the lignin-based biocatalyst from pine sawdust (see Figure 2a) shows a transmittance band between 1250–1160 cm^−1^, which stands for the S=O stretching vibrations of the SO_3_H group [12].

On the other hand, the maximum TAS for the lignin-based biocatalyst from processed wheat straw was 0.78 meq H^+^/g, achieved at 125 °C and 2 h. This TAS value is in the middle of that reported by Hu et al. [13], although they considered 110–150 °C and 20 h. Our results and those obtained for the pine sawdust are compared with those available in the state of the art form of a Table (see Table 4). Our experimental results show that the reaction time of the sulfonation reaction might be reduced drastically to achieve similar results for the TAS. Besides, the temperature of the sulfonation reaction can also be reduced. The FTIR spectra of the lignin-based biocatalyst from wheat straw (see Figure 2b) is more specific than that shown in Figure 2a and related to the biocatalyst from pine sawdust because it shows two peaks: At 1032 cm^−1^ and at 1162 cm^−1^, which are usually related to the S=O symmetric and asymmetric stretching vibrations, respectively [12].

Finally, it is important to recall that the Box-Behnken design has been applied to the TAS as the response variable. These values were computed via NaOH titration and, as a consequence, they might contain some relatively weak acid sites that are not responsible for the esterification of oleic acid. Thus, our research efforts are now directed to determine the acid strength of both biocatalysts. At the moment, we are assuming that such weak acid sites are relatively low. This is probably why both biocatalysts are able to satisfactorily conduct the esterification reaction of oleic acid in the presence of methanol, promoting yields that are close to those reported by Pua et al. [11] and Guo et al. [12]. Another pending issue regarding the characterization of the biocatalyst is the analysis of their reuse and stability.

## 4. Materials and Methods

### 4.1. Lignin Extraction

Pine sawdust was obtained from a local timber industry and wheat straw was provided by CINVESTAV-Guadalajara in the form of a residue from a saccharification-fermentation process (i.e., processed wheat straw). These wastes were prepared in accordance with the standard method, T264-cm-07, from the Technical Association of the Pulp & Paper Industry (TAPPI, Corners, GA, USA). Briefly, in this method the wastes are extracted successively with acetone and hot water [19]. Then, acid-insoluble lignin from both prepared wastes was recovered following the standard method, T222-om-02. As stated, this study aims to valorize the residual lignin from lignocellulosic wastes that have been processed in a biochemical platform scheme. Thus, we have decided to apply the method, T222-om-02, because it has basically the same objective to that of a traditional biochemical platform scheme: To degrade hemicellulose and cellulose and to discard lignin [20].

The acid-insoluble lignin from sawdust was immersed in 86% H_3_PO_4_ (1:1.3 lignin to acid ratio) and left for 1 h at room temperature. Then, the slurry was pyrolyzed at 400 °C for 1 h under N_2_ gas flow (2 L/min) in a 1100 °C muffle furnace (Terlab, TE-M20D, Mexico City, Mexico). The resulting sample was abundantly washed with distilled water and then dried [11]. The pretreatment with H_3_PO_4_, the subsequent pyrolysis and the final washing were also applied to the lignin from wheat straw.

### 4.2. Optimization of Sulfonation Rections

The pyrolized lignins were transformed into solid acid biocatalysts by sulfonation reactions using concentrated H_2_SO_4_ and following a Box-Behnken experimental design, with the previously reported conditions of Pua et al. [11] and Guo et al. [12] as references. These conditions were: A concentrated H_2_SO_4_ to lignin (mL/g) ratio of 10:1, temperature of 200 °C, and time of 2 h. However, we hypothesized that these operational conditions may be improved for cost–energy savings. Then, the abovementioned three factors were tested with lower values, as shown in Table 1. For the experimental design, these factors were labelled as follows: X_1_: The ratio of concentrated H_2_SO_4_ to lignin (mL/g), X_2_: Temperature (°C), and X_3_: Time (h). The sulfonation reactions were conducted by duplicate. The resulting samples from each experimental assay were washed with distilled water and then dried. The Total Acid Sites density (TAS, in meq H^+^/g) was computed for each sample using acid-base back titration [21,22], and the obtained values were recorded as the response variable for the proposed experimental design. Then, an optimization procedure was carried out using the statistical software, Statgraphics Centurion XVI.

### 4.3. Global Yield of Biocatalyst Synthesis

The yields related to the lignin extraction process, the pyrolysis process and the sulfonation processes were computed as follows:

(3) $$\mathrm{Lignin}\;\mathrm{extraction}\;\mathrm{yield}\;=\;100\;\times \;\left(\frac{\mathrm{mass}\;\mathrm{of}\;\mathrm{extracted}\;\mathrm{lignin}}{\mathrm{mass}\;\mathrm{of}\;\mathrm{waste}\;\left(\mathrm{sawdust}\;\mathrm{or}\;\mathrm{wheat}\;\mathrm{straw}\right)}\right)$$

(4) $$\mathrm{Pyrolysis}\;\mathrm{yield}\;=\;100\;\times \;\left(\frac{\mathrm{mass}\;\mathrm{of}\;\mathrm{pyrolized}\;\mathrm{lignin}}{\mathrm{mass}\;\mathrm{of}\;\mathrm{extracted}\;\mathrm{lignin}}\right)$$

(5) $$\mathrm{Sulfonation}\;\mathrm{yield}\;=\;100\;\times \;\left(\frac{\mathrm{mass}\;\mathrm{of}\;\mathrm{sulfonated}\;\mathrm{lignin}}{\mathrm{mass}\;\mathrm{of}\;\mathrm{pyrolyzed}\;\mathrm{lignin}}\right)$$

Then, to show how much biocatalyst can be obtained from a pine sawdust or wheat straw sample, the global yield of biocatalyst synthesis, as the product of the lignin extraction yield, the pyrolysis yield and the sulfonation yield (expressed as fractions), was determined, which can be expressed as follows:

(6) $$\mathrm{Global}\;\mathrm{yield}\;\mathrm{of}\;\mathrm{biocatalyst}\;\mathrm{synthesis}\;=\;100\;\times \;\left(\frac{\mathrm{mass}\;\mathrm{of}\;\mathrm{sulfonated}\;\mathrm{lignin}}{\mathrm{mass}\;\mathrm{of}\;\mathrm{waste}\;\left(\mathrm{sawdust}\;\mathrm{or}\;\mathrm{wheat}\;\mathrm{straw}\right)}\right)$$

### 4.4. Activity Test of the Biocatalysts

The combination of factors X_1_ X_2_, X_3_, which maximizes the TAS, was considered to synthesize the biocatalysts that were characterized via FTIR (Thermo Scientific, Nicolet iS5, Waltham, MA, USA) and whose activity was monitored in the esterification reactions of oleic acid with methanol [23]. The esterification reaction has been selected to test the biocatalysts because it plays an important role in the transformation of highly acidic oil samples into biodiesel. The operating conditions of the esterification reactions were: 5 h, 60 °C and the following oleic acid-methanol-biocatalyst mass ratio of 1:2.35:0.2. The reaction yield was computed, considering the percentage of free fatty acids in oleic acid and in the oily phase of the final esterification product, as suggested by Cavalcanti et al. [24]. The percentages of free fatty acids were determined using the American Society for Testing and Materials standard method D974-80 (ASTM D974-80).

## 5. Conclusions

The transformation of lignin from pine sawdust and processed wheat straw into a biocatalyst via a sulfonation reaction was optimized using a Box-Behnken experimental design whose factors were X_1_: The ratio of concentrated H_2_SO_4_ to lignin (mL/g), X_2_: Temperature (°C), and X_3_: Time (h) affecting the TAS, which was considered as a response variable in the design. Our experimental results show that the TAS for both biocatalysts can be maximized if the sulfonation reaction time is 1 h, in the case of the pine sawdust, and 2 h, in the case of the processed wheat straw. These operation times are significantly lower than those reported in the literature. Thus, these findings might imply cost and energy reductions if they are further considered for the synthesis of the abovementioned biocatalysts.

## 6. Patents

The results concerning the optimization of the lignin-based biocatalyst synthesis from pine sawdust are patent-pending in MEXICO (MX/a/2016/011129).

## Figures and Tables

**Table 1:** Box-Behnken experimental design results for sulfonation reaction in biocatalysts synthesis.

Ratio (mL/g) of Concentrated H_2_SO_4_ to Lignin (X_1_)	Temperature (°C) (X_2_)	Time (h) (X_3_)	Total Acid Sites (TAS) (meq H^+^/g)	
			Pine Sawdust	Wheat Straw
6:1	100	1.5	0.97 ± 0.13	0.68 ± 0.13
6:1	150	1.0	1.02 ± 0.06	n.d.
6:1	150	2.0	2.01 ± 0.06	0.59 ± 0.19
6:1	200	1.5	1.37 ± 0.25	0.45 ± 0.13
8:1	100	1.0	0.91 ± 0.13	n.d.
8:1	100	2.0	1.42 ± 0.00	0.77 ± 0.06
8:1	150	1.5	0.94 ± 0.13	0.63 ± 0.19
8:1	200	1.0	1.96 ± 0.06	0.58 ± 0.13
8:1	200	2.0	1.53 ± 0.13	0.58 ± 0.06
10:1	100	1.5	1.21 ± 0.25	0.67 ± 0.06
10:1	150	1.0	1.10 ± 0.13	0.77 ± 0.25
10:1	150	2.0	0.98 ± 0.06	0.82 ± 0.19
10:1	200	1.5	1.44 ± 0.19	0.60 ± 0.06

n.d. = not determined.

**Table 2:** ANOVA for pine sawdust biocatalyst.

Source	Sum of Squares	df	Mean Square	F-Ratio	*p*-Value
A:X_1_	0.1024	1	0.1024	2.84	0.1094
B:X_2_	0.7921	1	0.7921	21.94	0.0002
C:X_3_	0.225625	1	0.225625	6.25	0.0223
AC	0.621612	1	0.621612	17.22	0.0006
BB	0.261446	1	0.261446	7.24	0.0149
BC	0.437112	1	0.437112	12.11	0.0027
CC	0.332231	1	0.332231	9.20	0.0071
Total error	0.649729	18	0.036096		
Total (corr.)	3.28665	25			

R-squared = 80.23%, R-squared (adjusted by d.f.) = 72.54%.

**Table 3:** ANOVA for wheat straw biocatalyst.

Source	Sum of Squares	df	Mean Square	F-Ratio	*p*-Value
A:X_1_	0.0466617	1	0.0466617	26.67	0.0001
B:X_2_	0.0647717	1	0.0647717	37.01	0.0000
C:X_3_	0.000127019	1	0.000127019	0.07	0.7911
AB	0.0128	1	0.0128	7.31	0.0156
BB	0.0137366	1	0.0137366	7.85	0.0128
BC	0.000725595	1	0.000725595	0.41	0.5287
CC	0.0128658	1	0.0128658	7.35	0.0154
Total error	0.0279983	16	0.00174989		
Total (corr.)	0.2362	23			

R-squared = 88.15%, R-squared (adjusted by d.f.) = 82.96%.

**Table 4:** Comparative table of sulfonation conditions and total acid sites for the lignin-based biocatalyst.

Raw Material	Sulfonation Conditions	Total Acid Sites of Lignin-Based Catalyst	Reference
Kraft lignin	Temperature: 200 °C Time: 2 h H_2_SO_4_ to biomass ratio: 10:1 (g/mL)	2.21 mmol/g	Pua et al. [11]
Lignin from *Xanthoceras Sorbifolia*	Temperature: 150 °C Time: 2 h H_2_SO_4_ to biomass ratio: 5:10 (g/mL)	1.71 mmol/g	Guo et al. [12]
Lignin based activated carbon fibers	Temperature: 110 °C or 150 °C Time: 20 h H_2_SO_4_ to biomass ratio: 5:10 (mg/mL)	0.3–2.43 mmol/g	Hu et al. [13]
Mason pine alkali lignin	Temperature: 180 °C Time: 12 h H_2_SO_4_ to biomass ratio: 20:1 (g/mL)	1.46–3.52 mmol/g	Zhu et al. [14]
Lignin from 40–60 mesh pine powder (*Pinus tabuliformis*)	Temperature: 50 °C Time: 5 h Sulfuryl chloride+tetrachloroethane to biomass ratio: 75:10 (g/mL)	2.22 mmol/g	Liang at al. [17]
Alcell lignin	Temperature: 150 °C Time: 12 h H_2_SO_4_ to biomass ratio: 50:1 (g/mL)	1.86 mmol/g	Adhikari et al. [18]
Lignin from pine sawdust	Temperature: 200 °C Time: 1 h H_2_SO_4_ to biomass ratio: 10:1 (g/mL)	1.68 mmol/g	This study
Lignin from processed wheat straw	Temperature: 125 °C Time: 2 h H_2_SO_4_ to biomass ratio: 10:1 (g/mL)	0.74 mmol/g	This study
